# Supplementary material for: Nanobody generation and structural characterization of Plasmodium falciparum 6-cysteine protein Pf12p
Source: Biochem J. 2021 Feb 10;478(3):579–95. doi: 10.1042/BCJ20200415 (PMC7886318; doi:10.1042/BCJ20200415)

Supplementary Fig S1. BLI-affinity measurements with immobilized nanobodies and Pf12p D1D2 in solution. **(A)** Representative binding curves of six different Pf12p D1D2 concentrations to immobilized nanobodies are shown and were fitted to a 1:1 binding model. Pf12p D1D2 concentrations from 6 – 200 nM was used for all nanobodies except A10 which used Pf12p D1D2 concentrations from 13 – 400 nM. Corresponding K_D values are indicated. **(B)** Table containing determined kinetic and affinity data from three independent experiments showing the mean and standard error of the mean (SEM).

Supplementary Fig S2. Amino acid sequence alignment of Pf12p with orthologs of different *Plasmodium species*. The program ClustalO was used for the alignment [56]. The *Plasmodium* species with uniprot ID of the corresponding P12p protein is indicated on the left hand side of the alignment. Conserved cysteine residues are highlighted in yellow and the asparagine-rich region of Pf12p is highlighted in purple.

Supplementary Fig S3. Interdomain interactions of Pf12 D1-D2, Pf41 D1-D2 and Pf12p D1-D2. Interdomain linker regions are highlighted in green and sidechain residues at the interface of the two domains, D1 and D2, are shown in ball and stick representation. In all three structures the domain-domain contacts are mostly formed between connecting loops of D1 and the five-stranded β-sheet of D2. **(A)** Pf12, **(B)** Pf41, **(C)** Pf12p.

Figure S1

A

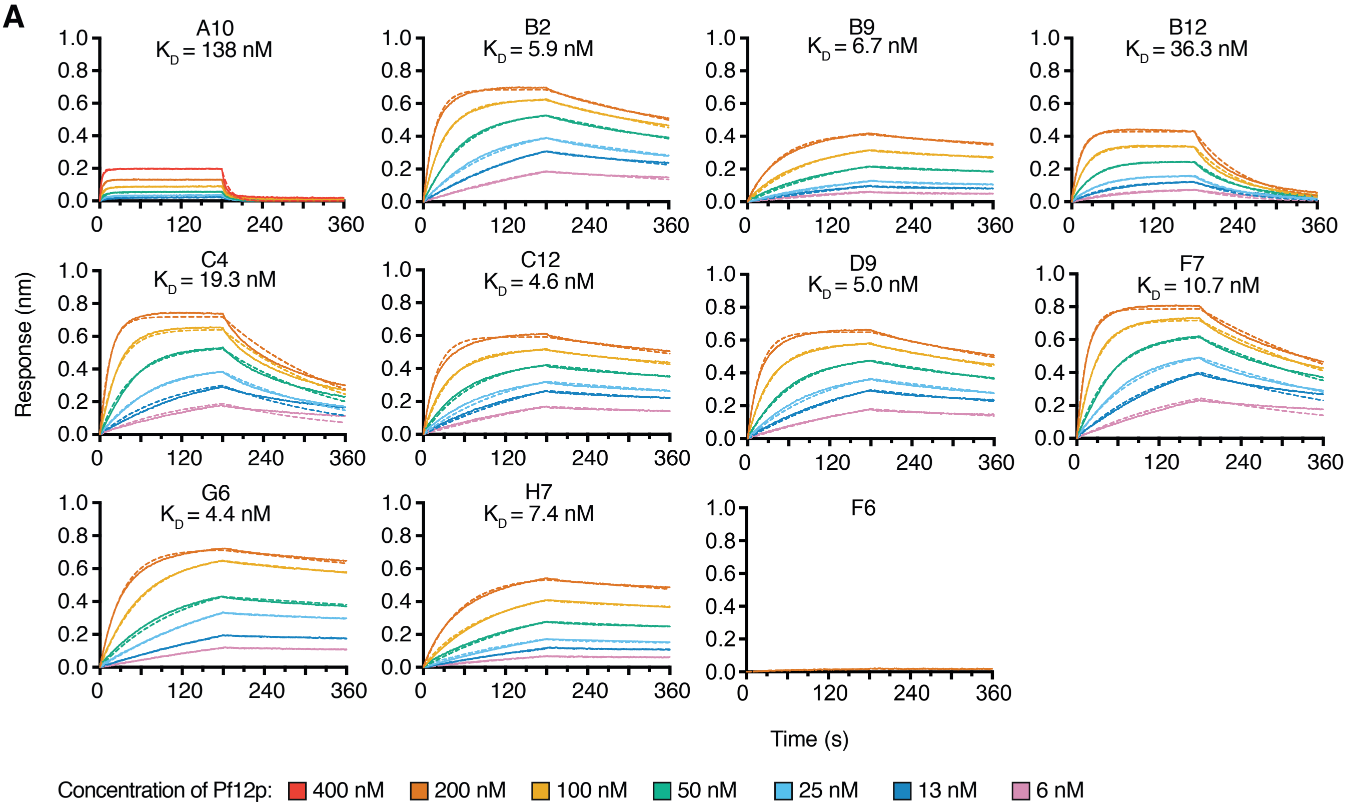

B

mAb	K_D (nM)	k_a (x 10^5 $M^{-1}s^{-1}$)	k_d (x 10^{-3} s^{-1})
A10	105.10 (± 24.89)	7.46 (± 0.43)	76.20 (± 14.98)
B2	6.76 (± 0.75)	3.00 (± 0.57)	1.95 (± 0.21)
B9	7.48 (± 0.88)	1.21 (± 0.27)	0.86 (± 0.13)
B12	25.27 (± 5.67)	5.38 (± 0.66)	12.87 (± 1.24)
C4	17.80 (± 2.73)	4.62 (± 1.73)	7.27 (± 1.56)
C12	3.96 (± 0.77)	2.68 (± 0.37)	1.03 (± 0.21)
D9	5.19 (± 0.98)	2.96 (± 0.44)	1.45 (± 0.09)
F7	10.63 (± 1.33)	3.81 (± 0.88)	3.82 (± 0.40)
G6	3.22 (± 0.81)	2.23 (± 0.67)	0.61 (± 0.03)
H7	6.71 (± 0.50)	1.12 (± 0.23)	0.76 (± 0.19)

Figure S2

```
P.falciparum_C6KSX1     -MHIVSFII-----------FFFA------------------------LFFPISICYKIN 24
P.berghei_Q4Z5T0        -------------------------------------------------------MIQNI 5
P.knowlesi_A0A1Y3DHK6   -MRVRHFSFLRLFLLLSLLVYHLPVQKQRHRSIPSWKYPDE-GDDHPPQNAFSHMANQVK 58
P.reichenowi_A0A151LP59 -MHIVSFLI-----------FFFA------------------------LFFPISICYKIN 24
P.malariae_A0A1D3SN11   -MQLVIFAI---------------VQ----------------------FLYFFSFAYKTN 22
P.ovale_A0A1A8VY82      -MPVVKSEG---------------TQ-----------------------ESSRERVQKSK 21
P.chabaudi_A0A4V0K2Q1   MMKTYFWLA---------------VH-----------------------FFSSFWMIQNI 22
P.yoelii_A0A078K5Q3     MMRIYFWLA---------------MH-----------------------IFSSFWMIQNI 22
P.vivax_A0A1G4H080      -MRVGHFSFVRLLLLQSLLVQRLPVQKQQQSSIPARKHPEEEGGGPSPQNALAQVANQMK 59
                                                                                 :

P.falciparum_C6KSX1     GVCDFSSEGLSLLPEEKLD---------FSVSRNVDKLSDENNVRHCVHFSKGFEYLRFI 75
P.berghei_Q4Z5T0        EICDFSRGSLDVALMNNKILIDN--N-------LKEENYNDNNIKHCVIFTKGLEIFTFI 56
P.knowlesi_A0A1Y3DHK6   GICDFSRGPLNVSTTENEIVPLLVQAELHAGSAPLSDAHTDEPVQRCVQFTKGMEVLTFV 118
P.reichenowi_A0A151LP59 GVCDFSSEGLSLLPEEKLD---------FSASRNVDKLSDENNVRHCVHFSKGFEYLRFI 75
P.malariae_A0A1D3SN11   DDCDFTREPLNVAWNNRNNALAPVDM-------EDEPYDNDNNIKYCVKFTKGFEILTFI 75
P.ovale_A0A1A8VY82      GECDFTRGSLDVSRSGNNKVAL---F-------EREEEAYDSNVKHCVKFTKGLEIMTFI 71
P.chabaudi_A0A4V0K2Q1   EICDFSKESLDVALTKDKSVIGN--S-------SNEENHSDNNIKHCVKFTKGFEIFTFI 73
P.yoelii_A0A078K5Q3     EICDFSRDSLDVTLMNNKIVIDN--N-------LKEENYNDNNIKHCVKFTKGLEIFTFI 73
P.vivax_A0A1G4H080      GTCDFSRAPLNVSCSENEIVALPGGEGAM-VSGTTGSTANDERARHCVQFTKGFDVLTFV 118
                          ***:   *.:                            :.  : ** *:**:: : *:

P.falciparum_C6KSX1     CPMRK--DNYEGIEIRPVECFEYIHI-EGREHKLSEILKGSLYEKSINDNIMTRDVFIPP 132
P.berghei_Q4Z5T0        CPKGNNNDNYKGVEIRPEQCFEKVRI-NGKEENLKDILKGVVIEKKETDTEIIRKALIPP 115
P.knowlesi_A0A1Y3DHK6   CPKRNT-EDYIGVEIRPMECFEKVRMHNGNKKKLNNVLKGVQLENIDTDSLSIRKVFIPP 177
P.reichenowi_A0A151LP59 CPMRK--DNYEGIEIRPVECFEFVRI-EGREQKLGEILKGSLYEKSINDNIMTRDVFIPP 132
P.malariae_A0A1D3SN11   CPKKG--INYEGIEMRPKECFEKIRI-NGRDENFTEILKGSIFESSETDSIIIRRVFIPP 132
P.ovale_A0A1A8VY82      CPKRS--IHYEAIEIRPTDCFVKVRV-NGIEERLNDFMKGVIFESRENDLSNIRKVFIPP 128
P.chabaudi_A0A4V0K2Q1   CPKGNNNDNYNGIEIRPVQCFEKVRI-NGKEENLKDVLKGVITENKETDTSIIRKAFIPP 132
P.yoelii_A0A078K5Q3     CPKGNNNDNYKGIEIRPEQCFEKVRI-NGKEENLKDILKGVIIEKKETDTEIIRKAFIPP 132
P.vivax_A0A1G4H080      CPKRSS-EDYSGVEIRPMSCFETVRRTDGTNQQLSEVLKGVQLENRDTDLLSIRRVFIPP 177
                        **      .* .:*:** .**  ::  :* ...: :.:**   *.  .*    * .:***

P.falciparum_C6KSX1     TIYEDMFFECTCDNSLTFKNNMIGIRGIMKIHLKKNILYGCDFDHDEKL----------- 181
P.berghei_Q4Z5T0        TIYQDMSFECSCDNSLTIKDNYIGARGIMKVHLKKNIIFGCDFNYDSNE----------- 164
P.knowlesi_A0A1Y3DHK6   TIYRNIIFECTCDNSLSFWNNKMGTRGIMRVHLRKNIVFGCDFDHRGGRENILEVEGELP 237
P.reichenowi_A0A151LP59 TIYEDMFFECTCDNSLTFKNNMIGIKGIMKIHLKKNILYGCDFDHDEKL----------- 181
P.malariae_A0A1D3SN11   TIYADMVIECTCDNSLTFKENFIGARGIMRVHLKRNKIFGCDFDSNIDGDDS-------- 184
P.ovale_A0A1A8VY82      TIYEDIVFECTCDNSLTFGDNQIGTRGIMRVHLKKNLVFGCDFDYDVMN----------- 177
P.chabaudi_A0A4V0K2Q1   TIYNDMSFECSCDNSLTIKDNTIGARGIMRVHLKKNKIFGCDFNYDASD----------- 181
P.yoelii_A0A078K5Q3     TIYQDMSFECSCDNSLTIKDNYIGARGIMKVHLKKNIIFGCDFNYDTNE----------- 181
P.vivax_A0A1G4H080      TIYQNFIFECSCDNSLTFWKNKMGARGIMRVHLRRNILFGCDFDHTGGVEYAGGLGGELP 237
                        *** :: :**:*****:: .* :* :***::**::* ::****:

P.falciparum_C6KSX1     -------------------------------------MKNKTAFTNFYDKQKILPLIGNN 204
P.berghei_Q4Z5T0        ----------------------------------PKLSNGKSAFAQFYDKQV-------- 182
P.knowlesi_A0A1Y3DHK6   AVDEAN----------RNTTGDWAFWRNAGPSAEELAERNKTAFSQFYTSEEV------- 280
P.reichenowi_A0A151LP59 -------------------------------------MKNKTAFTNFYDKQKILSLIDNN 204
P.malariae_A0A1D3SN11   -----TYG----RSDGGTRN----KCEGRLDNNCEESKSGRSAFVKYYDKSK-------- 223
P.ovale_A0A1A8VY82      -------------------------------------SRKRSAFVSFYEKNE-------- 192
P.chabaudi_A0A4V0K2Q1   ----------------------------------TKFSNGKSAFTNFYDNQA-------- 199
P.yoelii_A0A078K5Q3     ----------------------------------PKHSNGKSAFARFYDKII-------- 200
P.vivax_A0A1G4H080      MADEASVGSAEDRSAGGGSADDWAFWRNAGPSAEELAEKNKTAFTNFYPPGEV------- 290
                                                                ::**  :*

P.falciparum_C6KSX1     NNDDDNNDDDNNNDNNNNDNNNNNNNNNNNNNNNNNNNITCNVTIKKSQVYLGIICPDGY 264
P.berghei_Q4Z5T0        -------------------------------V-DSNKNIICNTQVNNKEVYLGLVCPEGY 210
P.knowlesi_A0A1Y3DHK6   -------------------------------NDAKDKGIICNVKITKREVYLGLVCPSGY 309
P.reichenowi_A0A151LP59 NNNNDDDDDD--------------------NNNNNNNNITCNVTIKQSQVYLGIICPDGY 244
P.malariae_A0A1D3SN11   -------------------------------I-RSNESITCNVTINKKEVYLGLICPEGY 251
P.ovale_A0A1A8VY82      -------------------------------I-KPGEEIVCNVKITKKEVYLGLICPEGY 220
P.chabaudi_A0A4V0K2Q1   -------------------------------I-DLNRSTVCNTEVNTKEVYLGLVCPEGY 227
P.yoelii_A0A078K5Q3     -------------------------------V-NSNKNIICNTQVNSKEVYLGLVCPEGY 228
P.vivax_A0A1G4H080      -------------------------------SLAKEKGLVCDVKITKREVYLGLVCPPGY 319
                                                            .   *:. :.  :****::** **

P.falciparum_C6KSX1     TLYPNDCFKNVIYDNNIIIPLKKI-IPHDILYHQDKNK--RITFASFTLNINENPPGFTC 321
P.berghei_Q4Z5T0        GMYPENCFENVLFEK-KVINITEL-IKHDVKLHIEKNK--NISFASFILNPNENPKSFSC 266
P.knowlesi_A0A1Y3DHK6   EMYPSNCFDRVLYKD-SIVRMSEL-IKHHVTFHMDSNR--RMSFATFSLDRNENPPGFTC 365
P.reichenowi_A0A151LP59 ILYPNDCFKNVIYDNNIIIPLKKI-IPHDILYHQDKNK--RITFASFTLNINENPPRFTC 301
P.malariae_A0A1D3SN11   TIYPSDCFENVLYEN-KIVNIKELLVSHDIKLHIDKKK--RMSFATFILNKNENPKGFSC 308
P.ovale_A0A1A8VY82      KMYPLTCFENVLHEK-EVVKINEL-VQHDVKLHMDVHK--QISFATFTLNKNENPNSFSC 276
P.chabaudi_A0A4V0K2Q1   EMYPENCFEYVLFES-NVVRINEL-IKHDVKLHIEKNKHTHMSFASFTLNPNENPKSFSC 285
P.yoelii_A0A078K5Q3     EIYPENCFENILFEN-KVIKISEL-IKHDIKLHIEKNK--NISFASFILNSNENPKSFSC 284
P.vivax_A0A1G4H080      EMYPSNCFERVLQKDGSIVRVNEL-LKHDVSFHADGNR--RMSFATFTLNRNENPQGFSC 376
                         :**  **. ::  ..  ::  :.:: : *.:  * : ::  .::**:* *: ****  *:*

P.falciparum_C6KSX1     YCIKDQTNINNPLIVNFHFSNQETSYATKNKNLFFYFIFIFPFLYVILLL 371
P.berghei_Q4Z5T0        HCIKNN-DNSFPLIANITFPIMNLIL------------------LISM-- 295
P.knowlesi_A0A1Y3DHK6   LCVRMDIPEAPPLQANFVYHNYESFGFHFRL-LYVLVVIL---LLVLC-L 410
P.reichenowi_A0A151LP59 YCIKDQTNINNPLIVNFHFSNQETSYATKNTNLFFYFILIFPFLYFILFL 351
P.malariae_A0A1D3SN11   QCVKNN-DNIFPLQANFEYANYESFSLCTHL-RYFVLLLS---LLVLF-L 352
P.ovale_A0A1A8VY82      HCVKDG-DHASILQANFTYANYESASLPVRL-ALLFLLPI---LFSLL-W 320
P.chabaudi_A0A4V0K2Q1   QCIKKN-ANAFPLIANIMFSNYESYSFNYHV-TYLILISI---ILISY-I 329
P.yoelii_A0A078K5Q3     HCIKNN-DNSFPLIANITFANYESYSFNFYA-TYFILIFI---FLISY-I 328
P.vivax_A0A1G4H080      MCLNVQAPEAPPLQANFAFHNYESAGVRFGL-PCALVALV---LLALC-L 421
                         *:.        * .*: :   :
```

Figure S3

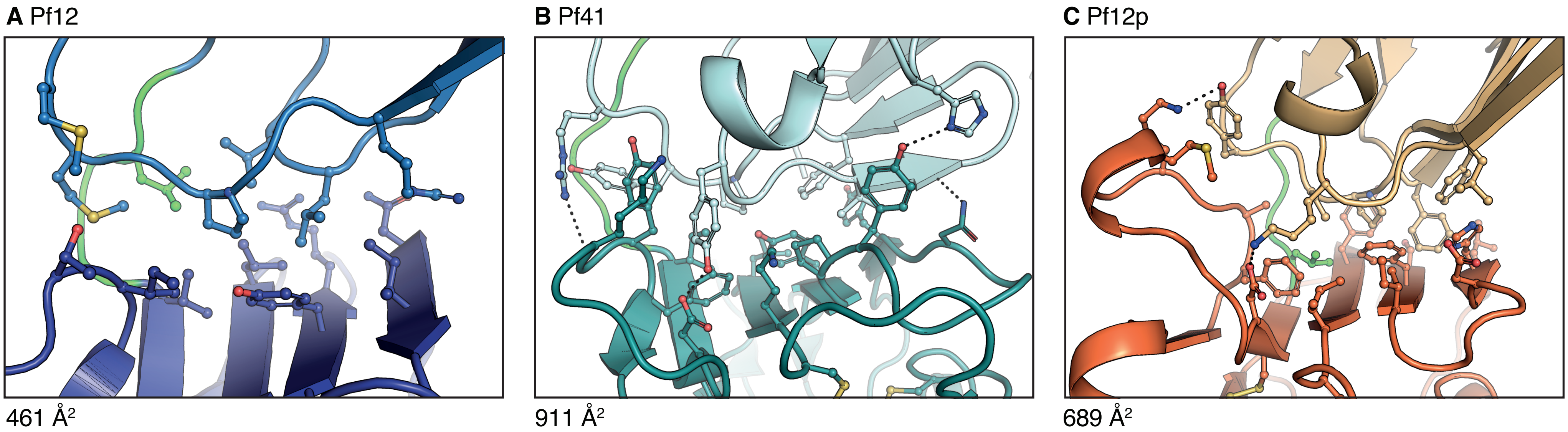

Supplement: Supplementary Figures S1-S3 [file BCJ-478-579-s1.pdf]
